# Anti-Tumor Effect of a Novel Soluble Recombinant Human Endostatin: Administered as a Single Agent or in Combination with Chemotherapy Agents in Mouse Tumor Models

**DOI:** 10.1371/journal.pone.0107823

**Published:** 2014-09-17

**Authors:** Zhihua Ren, Yanan Wang, Wenhong Jiang, Wei Dai, Yongping Jiang

**Affiliations:** 1 Biopharmagen Corp., Suzhou, China; 2 Department of Laboratory Diagnosis, Suzhou Municipal Hospital Affiliated Nanjing Medical University, Suzhou, China; 3 Biopharmaceutical R&D Center, Chinese Academy of Medical Sciences & Peking Union Medical College, Suzhou, China; 4 Department of Environmental Medicine, New York University Langone Medical Center, Tuxedo, New York, United States of America; Columbia University, United States of America

## Abstract

**Background:**

Angiogenesis has become an attractive target in cancer treatment. Endostatin is one of the potent anti-angiogenesis agents. Its recombinant form expressed in the yeast system is currently under clinical trials. Endostatin suppresses tumor formation through the inhibition of blood vessel growth. It is anticipated that combined therapy using endostatin and cytotoxic compounds may exert an additive effect. In the present study, we expressed and purified recombinant human endostatin (rhEndostatin) that contained 3 additional amino acid residues (arginine, glycine, and serine) at the amino-terminus and 6 histidine residues in its carboxyl terminus. The recombinant protein was expressed in *E. Coli* and refolded into a soluble form in a large scale purification process. The protein exhibited a potent anti-tumor activity in bioassays. Furthermore, rhEndostatin showed an additive effect with chemotherapy agents including cyclophosphamide (CTX) and cisplatin (DDP).

**Methods:**

rhEndostatin cDNA was cloned into PQE vector and expressed in *E. Coli*. The protein was refolded through dialysis with an optimized protocol. To establish tumor models, nude mice were subcutaneously injected with human cancer cells (lung carcinoma A549, hepatocellular carcinoma QGY-7703, or breast cancer Bcap37). rhEndostatin and/or DDP was administered peritumorally to evaluate the rate of growth inhibition of A549 tumors. For the tumor metastasis model, mice were injected intravenously with mouse melanoma B16 cells. One day after tumor cell injection, a single dose of rhEndostatin, or in combination with CTX, was administered intravenously or at a site close to the tumor.

**Results:**

rhEndostatin reduced the growth of A549, QGY-7703, and Bcap37 xenograft tumors in a dose dependent manner. When it was administered peritumorally, rhEndostatin exhibited a more potent inhibitory activity. Furthermore, rhEndostatin displayed an additive effect with CTX or DDP on the inhibition of metastasis of B16 tumors or growth of A549 tumors.

**Conclusion:**

Soluble rhEndostatin exhibits a potent anti-tumor activity in mouse xenograft models and it also has an additive effect with CTX and DDP, implying possible applications in clinical settings.

## Introduction

Angiogenesis plays a key role in promoting growth, invasion and metastasis of tumors [1], [2], [3]. It has become an attractive target for tumor therapy. Endostatin, a cleaved product of the carboxyl-terminal domain of collagen XVIII, was originally found in conditioned media from a murine endothelial tumor cell line (hemangio-endothelioma). It was reported that endostatin inhibits endothelial cell proliferation and migration both in vivo and in vitro, thus inhibiting tumor growth [4], [5]. Endostatin has been evaluated in clinical trials. In a Phase I clinical study, Endostatin was reported to be safe and well tolerated in advanced cancer patients, demonstrating some antitumor activities [6], [7]. Of the 74 patients' cohort, minor responses have been found in 2 patients (40% and 17% reduction in tumor volume for 22 and 11 months, respectively), and mixed responses in 2 patients for 14 or 5 months; In addition, 8 patients were reported to have stable disease for more than 3 months [8]. Furthermore, independent studies have shown that Endostatin decreases blood flow in tumors in a dose-dependent manner as measured by positron emission tomography scan [9], [10] and that it induces apoptosis in tumor cells [11], [12].

Endostatin that was used in previous clinical studies was expressed in yeast [13], [14], which makes the preparation costly and laborious. Endostatin has no direct cytotoxicity against tumor cells. The anti-tumor effect of endostatin stems from its ability to inhibit neo-angiogenesis around tumors, thus preventing tumor growth and progression. while it shows no direct cytotoxicity against tumor cells [15]. It has been reported that endostatin-mediated inhibition of angiogenesis was most effective when it is combined with other therapies, especially chemotherapy [16], [17], [18].

The conventional chemotherapy agents, such as cyclophosphamide (CTX) and cisplatin (DDP), directly target tumor cells and induce cytotoxicity. However, the efficacy of chemotherapy agents is often compromised due to their adverse side effects such as myelosuppression and development of drug resistance [19], [20]. It is believed that a combination of anti-angiogenesis agents and chemotherapy agents will significantly enhance the therapeutic efficacy and reduce side effects [17], [21]. In this study, we expressed rhEndostatin with additional arginine, glycine, and serine residues at the N-terminus using the prokaryotic expression system. The added amino acid residues were expected to enhance the binding affinity of target molecule to the cellular receptor [22]. rhEndostatin also contained 6-histidine residues at the caboxyl terminus for affinity purification. We also developed and optimized a protocol for efficient refolding of bioactive rhEndostatin in a large scale. We demonstrated that rhEndostatin exhibited a potent bioactivity and was capable of synergizing with CTX and DDP in suppression of tumor cell growth. Our combined studies strongly suggest that rhEndostatin can be developed as a therapeutical agent for the treatment of various malignancies.

## Materials and Methods

### Ethics Statement

All research involving animals was conducted according to relevant national and international guidelines. Male BALB/c nude mice, weight 16.0∼25.1 g were purchased from SLAC Company (Shanghai, China). The experiment protocols were approved by the Institutional Animal Care and Use Committees of Soochow University (IACUC permit number: SYXK(Su) 2012-0045), and were in accordance with the Guidelines for the Care and Use of Laboratory Animals (National Research Council, People's Republic of China, 2010). We further attest that all efforts were made to ensure minimal suffering.

### Cell lines and reagents

Human A549 lung carcinoma cell line [23], human umbilical vein endothelial cells (HUVEC) [4], [24], and mouse B16 melanoma cell line [25] were purchased from ATCC (Manassas, VA, USA). Human hepatocellular carcinoma cell line QGY-7703 [26], and human breast cancer cell line Bcap37 [27] were from Shanghai Institute of Cell Biology (Shanghai, China). Restriction enzyme BamHI and HindIII were purchased from New England Biolabs (Ipswich, MA, USA). The PQE60 plasmid, *E.Coli* M15 and Ni-NTA purification kit were obtained from QIAGEN (Valencia, CA, USA). Isopropyl-1-thio-ß- D-galactopyranoside (IPTG) was from Bio-Basic Inc (Amherst, NY, USA) to induce rhEndostatin expression in bacteria. Guanidine Hydrochloride was from Amresco Inc (Solon, OH, USA). Urea was from Shanghai Experimental Reagent Inc (Shanghai, China). M199 medium and RPMI 1640 medium were from Gibco (Grand Island, NY, USA). Fetal bovine serum was from Hyclone (Logan, UT, USA). Basic fibroblast growth factor (bFGF) and vascular endothelial growth factor (VEGF) were from R&D systems (Minneapolis, MN, USA). MTT was from Cell Biolabs (San Diego, CA, USA). DMSO was from Sigma Aldrich (St. Louis, MO, USA). Cisplatin (DDP) was purchased from Shandong Qilu Pharmaceutical Co., Ltd (Shandong, China), and Cyclophosphamide (CTX) was from Shanghai Hualian Pharmaceutical Co, Ltd (Shanghai, China).

### Sequence Modified rhEndostatin and Expression of rhEndostatin in *E.Coli*

Human endostatin mRNA was extracted from Chinese embryo hepatocytes and cDNA was obtained by reverse transcription-polymerase chain reaction (RT-PCR). The amplified products were fractionated on agarose gels, and then ligated to plasmid PQE60, after been digested with restriction enzyme BamHI and HindIII. Mutant rhEndostatin was made by adding CGAGGUUCC after the starting codon of AUG, and ligating CACCACCACCACCACCAC before the stop codon. The PQE60 plasmid expressing mutant rhEndostatin was transformed into *E.Coli* M15, in a form as inclusion body.

### rhEndostatin purification and refolding in large scale

The inclusion body was collected from IPTG induced bacteria lysate and dissolved with Guanidine Hydrochloride (6 Mol). Ni-NTA Purification System has been employed to purify polyhistidine-containing rhEndostatin as previously described [28]. The purified rhEndostatin protein were then diluted with a phosphate buffer containing 8 Mol of Urea (PH 4.5) to 0.3 mg/ml, and the refolding protein was obtained by a series of dialysis procedure with optimized buffer conditions at 4°C.

### rhEndostatin inhibits human umbilical vein endothelial cell proliferation in vitro

Primary human umbilical vein endothelial cells (HUVEC) at 2–5 passages were suspended in M199 basal medium supplemented with 10% heat-inactivated fetal bovine serum, and then plated in 96-well culture plates at 5×10^3^ cells per 100 µl/well. Cells were incubated with rhEndostatin at various concentrations (0.1, 0.2, 0.5, 1, 2, 5, 8, 16 µg/ml) in bFGF(5 ng/ml) supplemented M199 medium or basal medium alone at 200 µl volume for 72 hours at 37°C. 20 µl of MTT(5%) was added to each well at 4 hours before the end of treatment, and then aspirated carefully. Cells were further incubated with 100 µl of DMSO for 15 minutes on the shaker, and optical density (OD) value was read at 570 nm wave length with the microplate reader (Bio-Rad, Hercules, CA. USA).

### Inhibition of chick embryo chorioallantoic membrane (CAM) angiogenesis by rhEndostatin

Chick embryonic chorioallantoic membrane assay was employed for evaluating the anti-angiogenesis activity of rhEndostatin. All procedures were carried out in a biosafety hood under sterile conditions. Briefly, fertilized eggs were incubated for 7 days at 37°C and 60% humidity. A window was made on the top of each egg after 2 days' incubation. Chick embryonic chorioallantoic membrane was exposed by tearing up the egg membrane with a tip. A filter paper disc of 5 mm diameter containing 20 µl of rhEndostatin (0.3 mg/mL) or saline was gently implanted, and changed every day for 10 days. The membrane was cut around the air sac, which was turned upside down and observed and photographed by a stereomicroscope. The number of blood vessel branch points was counted, and the vessel density was calculated according to the size of area where the blood vessel distributed to evaluate the anti-angiogenesis activity.

### In vitro anti-tumor study on rhEndostatin

All tumor cell lines (human A549 lung carcinoma cell line and mouse B16 melanoma cell line, human hepatocellular carcinoma cell line QGY-7703, and human breast cancer cell line Bcap37) were maintained in RPMI 1640 supplemented with 10% FBS. Human umbilical vein endothelial cells were cultured in RPMI 1640 supplemented with 2% FBS and 10 ng/mL each of VEGF and b-FGF. In vitro activity assay on rhEndostatin was performed as O'Reilly MS et al. described [4], with minor modifications.

### Animals

Male BALB/c nude mice, at 4–6 weeks of age, with an average weight of 20.5±1.2 g (16.0∼25.1 g) were employed in our studies. A total of 50 mice were involved in each of the following anti-tumor studies with single rhED treatment, including hepatocellular carcinoma QGY-7703, A549 lung carcinoma, or Bcap37 breast cancer, which were further divided into 5 groups, randomly. To evaluate the combination effects with CTX or DDP, a total of 160 nude mice (N = 10 in each treatment group) were selected. Another 60 nude mice (N = 10 each) were used to analyze if local rhEndostatin delivery will improve the tumor inhibition rate, in A549 lung carcinoma and Bcap37 breast cancer models. Animals were housed in individual stainless steel cages in a SPF facility in Soochow University, with a regulated temperature of 24±2°C, relative humidity of 50±10%, and a 12-h light cycle. Animals were sacrificed by Carbon dioxide (CO_2_) inhalation at 24 hours post the final treatment. Tumor tissues and organs were collected after euthanasia.

### Tumor-bearing mouse studies

The inhibition capabilities of rhEndostatin to tumor growth and tumor metastasis were evaluated. The tumor-bearing mouse model was generated by injecting BALB/c strain nude mice subcutaneously with 6×10^6^/100 µl of each tumor cells, including human A549 lung carcinoma, human hepatocellular carcinoma QGY-7703 and human breast cancer Bcap37 cells. When the tumors volume were about of 170 mm^3^, approximately 15 days after implantation, either rhEndostatin alone, or in combination with DDP were delivered to the animal model either intravenously or subcutaniously at the site close to the inoculated tumor, to evaluate the inhibition rate of tumor growth. Whereas rhEndostatin alone or along with CTX was given intraperitoneally to observe the metastatic rate of tumor. The mice from control group were received injections of phosphate-buffered saline (PBS) at the same volume intravenously. The mice were evaluated for tumor growth at every other day. The formula, width^2^×length×0.52, was applied to estimate the volume of a spheroid as described [5]. The formula, [(tumor volume of control-tumor volume of treatment)/tumor volume of control]×100%, was used to calculate the inhibition rate of tumor growth [5]. For the estimation of the metastasis inhibition rate, tumor-bearing mice were sacrificed by carbon dioxide asphyxiation, and evaluation was calculated with following formula: [(numbers of tumor colonies in lung of control mice numbers of tumor colonies in lung of treatment mice)/numbers of tumor colonies in lung of control mice]×100%. All procedures involving animals were done in accordance with national and international laws and policies.

### Statistical analysis

Comparison of variables was performed using One-Way ANOVA in SPSS program (SPSS). A statistically significant difference was defined as *P*<0.05.

## Results

### Expression, refolding, and purification of rhEndostatin in *E. coli*

**Figure 1:**
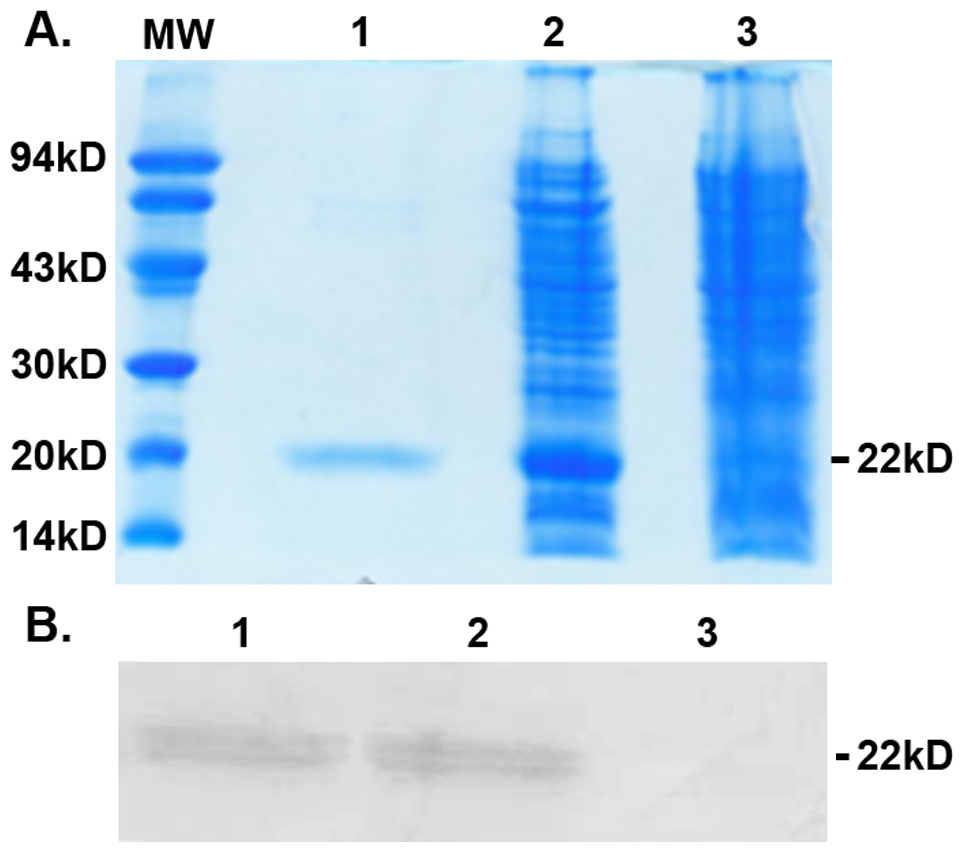
SDS-PAGE and immunoblotting of expression and purified rhEndostatin. A. SDS-PAGE of rhEndostatin. Lane 1: Pre-stained molecular weight markers; Lane 2: Purified rhEndostatin; Lane 3: Induced bacterial lysates; Lane 4: Non-induced bacterial lysates. (B). Immunoblotting of rhEndostatin. Lane 1: Purified rhEndostatin Lane 2: Whole bacterial cell lysates after IPTG induction; Lane 3: Non-induced bacterial lysates.

Modified rhEndostatin was expressed in *E. coli* using a pQE vector expression system. Following the addition of IPTG, rhEndostatin was highly induced (Figure 1A). Harvested from 40-L bio-reactor, about 1.5 g/L of rhEndostatin inclusion body was collected. The rhEndostatin was purified by affinity purification with nickel (Ni) resin followed by size-exclusion chromatography, and then the denatured protein underwent an optimized refolding procedure to gain a refolding rate of over 80%. Eventually, about 200 mg/L of purified rhEndostatin with bioactivity was generated. SDS-PAGE analysis revealed that rhEndostatin was purified to homogeneity and remained intact (Figure 1A). Immunoblotting showed that the commercial available rhEndostatin antibody detected IPTG-induced sequence-modified- rhEndostatin in the total bacterial lysates as well as its purified form (Figure 1B), suggesting that the amino acids addition did not significantly change the overall conformation of the protein. Protein sequencing analysis confirmed that the purified protein was the modified form of rhEndostatin with the addition of arginine, glycine, and serine residues at the amino-terminus (data not shown).

### Anti-angiogenesis assay on rhEndostatin in vitro and in vivo

**Figure 2:**
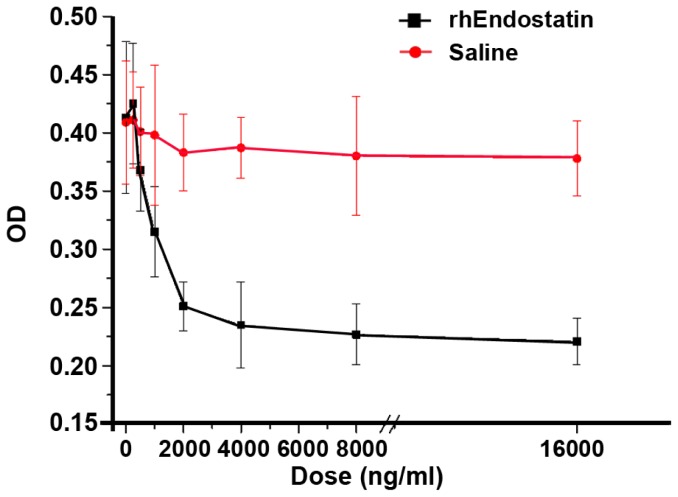
The inhibition effect of rhEndostatin on the growth of HUVEC *in vitro*. HUVECs were planted in fibronectin coated 96-well microtiterplates at 5×10^∧^3cells per well with bFGF supplemented M199 medium. rhEndostatin was applied at the dosage of 0.1, 0.2, 0.5, 1.0, 2.0, 4.0, 8.0, 16 µg/ml, as described before. All the treatments were triplicated. The OD value were shown as mean ± SD. Treatment with 2 µg/mL of rhEndostatin and above significantly inhibited HUVEC proliferation compared to saline vehicle control.

**Figure 3:**
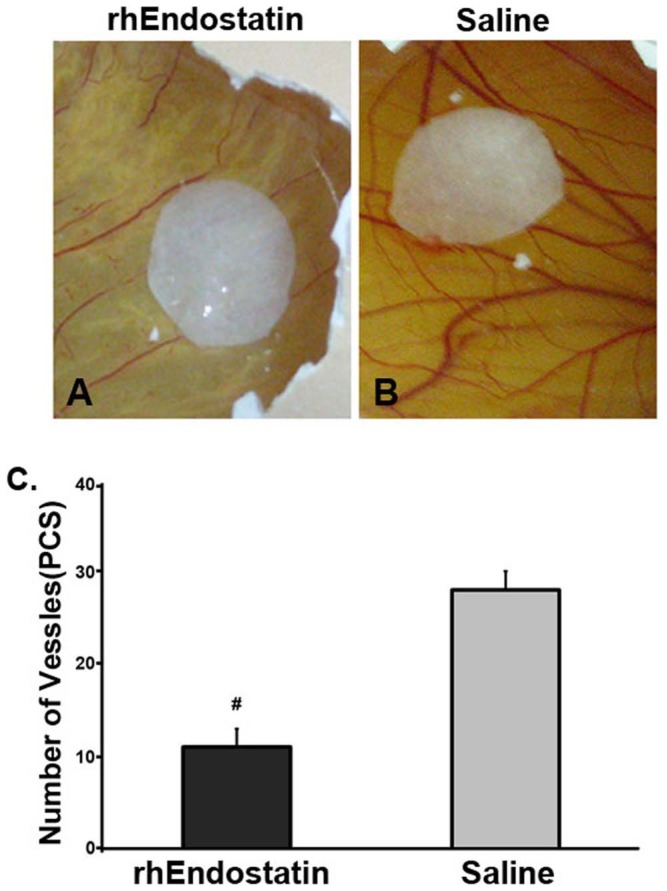
*In vivo* inhibition of angiogenesis using chicken embryo CAMs. Treated with rhEndostatin at 6 µg/disc (**A**), or treated with saline vehicle control disc (**B**) the vessels numbers were calculated(n = 8, each) in CAMs assay after 10 days treatment, and data were presented as mean±SD. **#** indicated *P*<0.05. (**C**).

To determine whether rhEndostatin could inhibit the growth of endothelial cells in vitro, HUVECs had been treated with rhEndostatin at the dose range from 0.1 to 16 µg/ml. Cell variability has been evaluated by MTT assay. rhEndostatin remarkably inhibited HUVEC proliferation in a dose depended manner (figure 2). rhEndostatin significantly inhibited HUVECs growth at the concentration above 0.2 µg/ml compared with the saline vehicle control. *In vivo* anti-angiogenic effect was investigated on a chicken embryonic chorioallantoic membrane assay (CAMs). CAMs vascularization was inhibited by rhEndostatin at 6 µg/disc for 10 days incubation. Figure 3B showed a normal looking of vascularization of untreated CAMs with primary, secondary and tertiary vessels and dendritic branching. On the contrary, CAMs treated with rhEndostatin demonstrated a distorted vasculature or the absence of blood vessels (Figure 3A).

### Antitumor efficacy of rhEndostatin administered as a single agent

**Figure 4:**
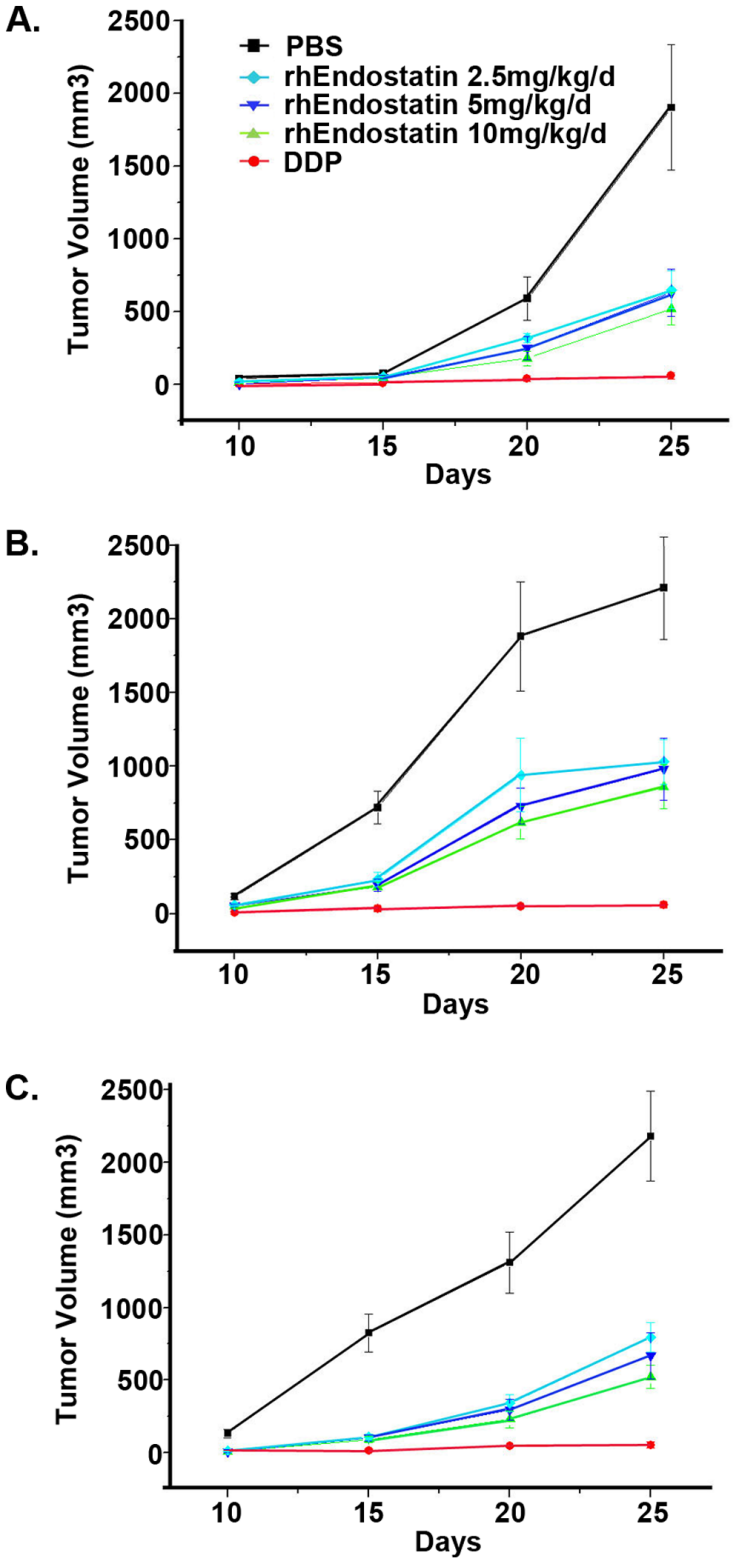
Inhibition of human tumor growth by administrating rhEndostatin intravenously. Hepatocellular carcinoma QGY-7703(A), A549 lung carcinoma(B), or Bcap37 breast cancer cells(C), were implanted to nude mice at the dosage of 6×10^6^ cells each, subcutaneously. Tumor-bearing mice were treated with either DDP (2 mg/kg/day) for successively 7 days(•), or rhEndostatin 10 mg/kg/day(▴), rhEndostatin 5 mg/kg/day(▾), and rhEndostatin 2.5 mg/kg/day(□) for successively 15 days. PBS(▪) was injected as the vehicle control. Results were presented as mean±SD (n = 10, each). Mice administered with rhEndostatin i.v. exhibited a significant reduction in tumor volume, compared with that in vehicle control group.

**Figure 5:**
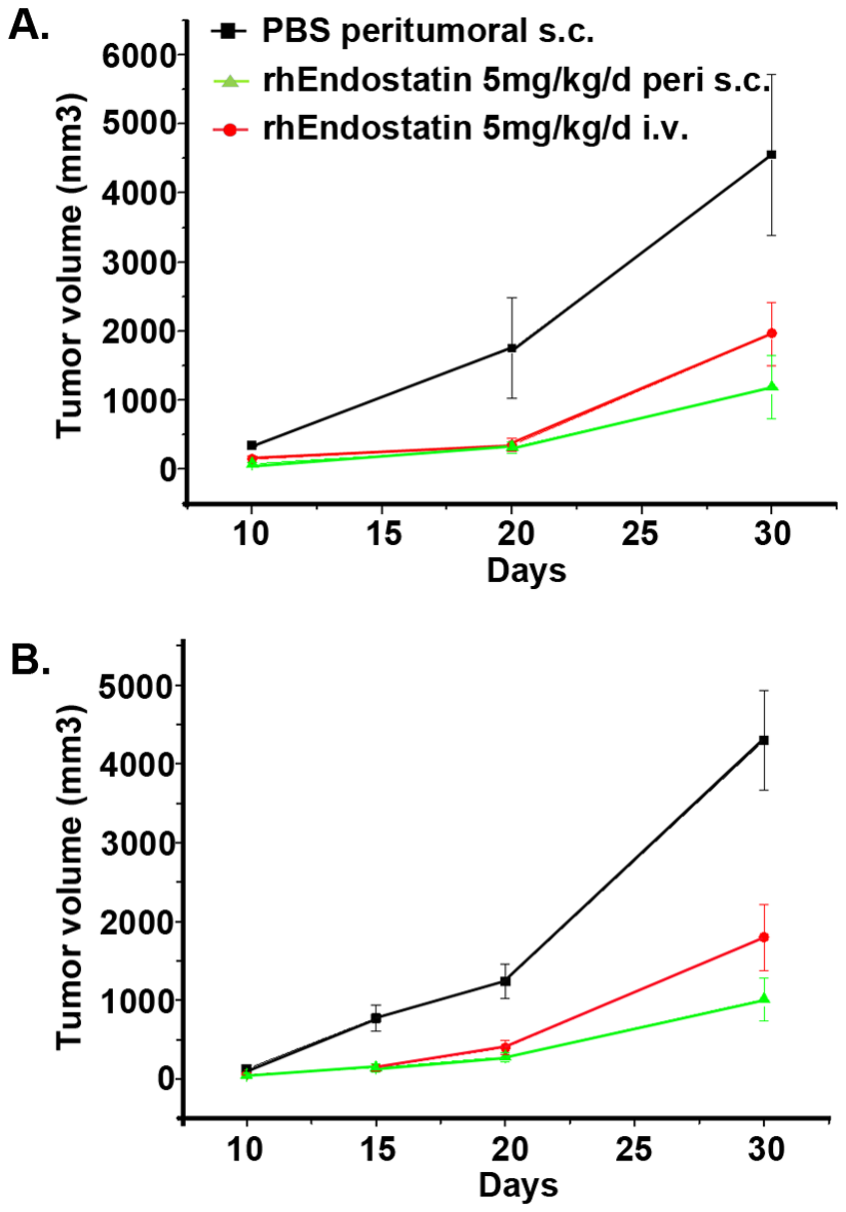
Inhibition of human tumor growth by administrating rhEndostatin s.c. peritumorally. Either A549 lung carcinoma cells (A), or Bcap37 breast cancer cells(B) were implanted to nude mice of 6×10^6^ tumor cells each, subcutaneously. Tumor-bearing mice were then treated for successively 15 days with rhEndostatin (5 mg/kg/day) intravenously (•), or at the site close to the tumor (▴). PBS was injected as the vehicle control at the site close to the tumor (▪). Results were presented as mean±SD(n = 10). Tumor volume was measured every other day, and the data indicated an enhanced reduction in tumor volume, when administered rhEndostatin s.c. peritumorally.

We first studied the anti-tumor bioactivity of rhEndostatin both in vitro and in vivo. rhEndostatin had no effect on the growth of any kind of tumor cells in vitro (data not shown). In the *in vivo* study, tumor cells were implanted subcutaneously, and measurable solid tumors were developed approximately 14 days post implantation at the injection site in all mice. Administration of rhEndostatin alone significantly delayed the growth of human A549 lung carcinoma, human hepatocellular carcinoma QGY-7703 and human breast cancer Bcap37 (Figure 4A, B, C), by 45% -35% at a high to medium dosage, compared with PBS vehicle control. Moreover, subcutaneous administration of rhEndostatin peritumorally exhibited a higher inhibition rate on day 30 post treatment than that by intravenous injection (Figure 5). Endostar, a version of commercially available endostatin, also exhibited an inhibitory activity toward A549 xenograft tumors. Although no statistically significant difference was observed between Endostar and rhEndostatin in blocking tumor growth, on day 9 rhEndostatin appeared to be more efficacious in reducing the tumor size (Figure S1).

### rhEndostatin administered in combination with CTX

**Figure 6:**
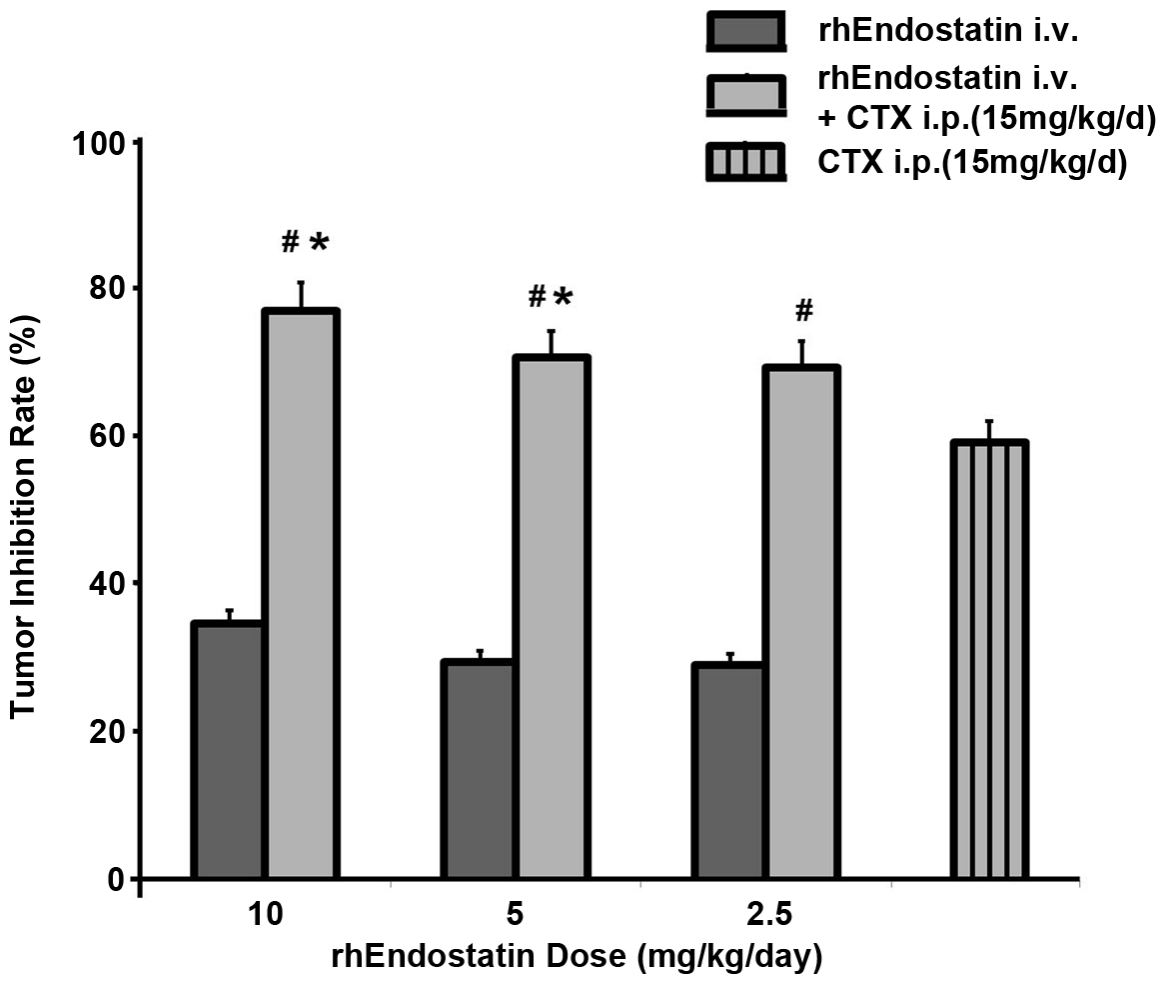
Additive effect of rhEndostatin with CTX on the metastasis of B16 melanoma. Nude mice (n = 10, each) were inoculated with 2.5×10^5^ tumor cells intravenously to establish a metastasis model. Mice were either administered i.v. with rhEndostatin alone at 10 mg/kg/day, 5 mg/kg/day, or 2.5 mg/kg/day for 15 days successively, or combined treated with CTX at the dosage of 15 mg/kg/d for successively 7 days, then intravenously administered with rhEndostatin at 10 mg/kg/day, 5 mg/kg/day, or 2.5 mg/kg/day for successively 15 days. The mice were sacrificed to count the metastasis colony in the lung on day 25 post inoculation. The formula, [(mean colony of PBS control- mean colony of treated mice)/mean colony of PBS control]×100, was used to calculate the inhibition rate of metastasis. #: *P*<0.05 vs mice administered with single rhEndostatin treatment at the same dose, respectively; *: *P*<0.05 vs mice treated with CTX alone, at the dosage of 15 mg/kg/day.

B16 melanoma metastasis model was employed to assay the additive effect of rhEndostatin with CTX. As shown in Figure 6, intravenously administration of three dosages of rhEndostatin (10 mg/kg/day, 5 mg/kg/day, and 2.5 mg/kg/day, respectively) for successive 15 days significantly inhibited the metastasis of B16 melanoma, with the inhibition rate of 34.87%, 29.66% and 29.26%, respectively. Furthermore, rhEndostatin and CTX exhibited an additive effect. CTX (15 mg/kg/day) administered alone in the first 7 days inhibited the metastasis by 59.32%, whereas rhEndostatin, which was intravenously injected at the dosage of 10 mg/kg/day, 5 mg/kg/day, and 2.5 mg/kg/day for successive 15 days, increased the inhibition rate to 77.15%, 70.94%, and 65.93%, respectively.

### rhEndostatin used in combination with DDP

**Figure 7:**
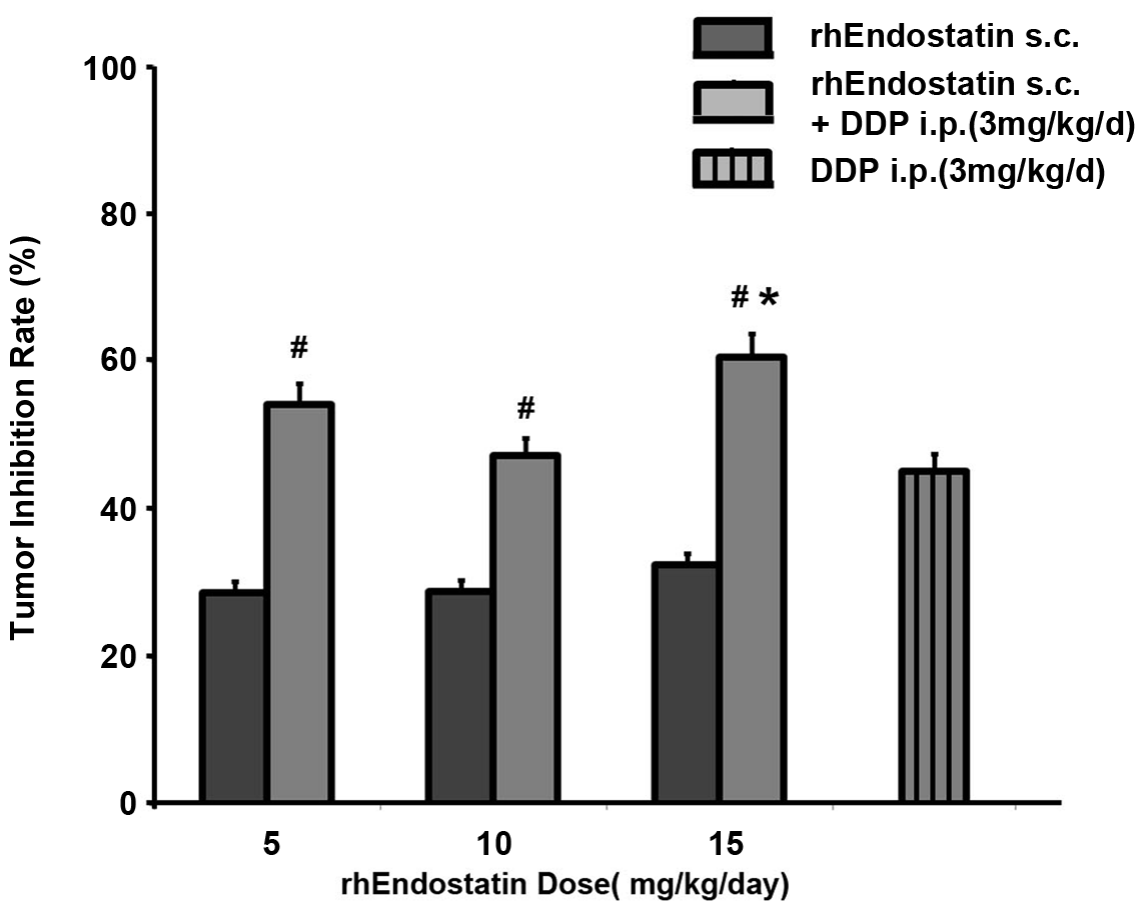
Additive inhibition effect of rhEndostatin with DDP on the growth of A549 lung cancer. Nude mice (n = 10, each) were inoculated with 6×10^6^ tumor cells subcutanously to establish the tumor-bearing model. The animals were either administered peritumorally with rhEndostatin alone at 5 mg/kg/day, 10 mg/kg/day, and 15 mg/kg/day for 15 days, successively, or combined with DDP treatment at 3 mg/kg/d every other day for 15 days. The tumor volume was measured every other day, and the formula, [(tumor volume of control-tumor volume of treatment)/tumor volume of control]×100%, was used to calculate the inhibition rate of tumor growth. #: *P*<0.05 vs mice administered with single rhEndostatin treatment at the same dose, respectively; *: *P*<0.05 vs mice treated with DDP alone at 3 mg/kg/day.

A nude mouse model with xenograft tumors (A549 cells) was used to evaluate the additive effect of rhEndostatin with DDP. As shown in Figure 7, three dosages of rhEndostatin, 5 mg/kg/day, 10 mg/kg/day, and 15 mg/kg/day, were administered peritumorally, twice a day for successive 15 days. Significant inhibition of tumor growth was observed with the inhibition rate of 28.79%, 28.88% and 32.43% (N = 10, each), respectively. Furthermore, an additive effect was also exhibited when rhEndostatin administered in combined with DDP. DDP (3 mg/kg/day) administered alone by i.p. in the first 7 days suppressed the tumor volume by 45.19%. After the first 7 days treatment, DDP (3 mg/kg/day), was administered in combination with rhEndostatin at the dose of 5 mg/kg/day, 10 mg/kg/day, and 15 mg/kg/day, respectively, for another 15 days successively. The inhibition rate was increased to 54.24%, 47.26%, and 60.66%, respectively.

## Discussion

Angiogenesis is critical for growth, invasion and metastasis of solid tumors. Most solid tumors begin as avascularized malignant cells [29], [30], [31], [32]. Without further vascularization, solid tumors cannot grow beyond 1–2 mm^2^ [33]. And there is a close correlation between microvessel counts and metastatic cancer. The mean microvessel count within biopsy tissue for the metastatic group is 76.8 vessels per field compared with 39.2 for those without metastasis (*P*<0.0001) [34]. Endostatin is first reported by O'Reilly MS as a potent angiogenesis inhibitor [35]. Several studies subsequently reported the anti-angiogenesis and anti-tumor activity of endostatin [4], [5], [36], [37]. Most of them employed endostatin which expressed in eukaryotic cells, or administered directly in the form of inclusion body, which leads to a limited yield rate as well as a low antitumoral bioactivity. The yeast expression system for the generation of rhEndostatin is characterized as a relative high cost, a low yield (with 10–20 mg/L of soluble protein produced [38]), and an unstable structure with a common feature of 2–4 amino acid residues truncated from the N-terminus of rhEndostatin. These shortcomings have limited the application of rhEndostatin in the clinical practice. The reported *E. Coli* expression system for rhEndostatin, on the other hand, has several salient features including low cost and a high expression rate; however, the refolding efficiency was low (less than 1% [39]). Therefore, our strategy focused on the optimization of refolding and purification procedure, in order to provide a sufficient amount of soluble form of rhEndostatin which can be potentially used for clinical trials. Our current study is among the very few that use rhEndostatin expressed in *E. Coli* and refolds into a soluble form with an optimized dialysis procedure (patent pending), which yields about 100 mg/L of soluble protein with a purity of 99%. Structural integrity of rhEndostatin is evaluated by protein sequencing, and anti-angiogenic and antitumor activities are also determined, which yields a comparable result as compared with the commercially available Endostar.

In order to achieve an optimal tumor inhibition and regression, researchers have demonstrated that it is critical to continuously elevate endostatin levels in blood circulation [40], [41]. A short half-life of 2 h of endostatin has been reported, which requires a more frequent drug administration in addition to escalating drug doses to achieve the effective plasma concentration. Our modified rhEndostatin contains 3 additional amino acid residues (arginine, glycine, and serine) at the amino-terminus, which appears to increase the overall positive charge of protein, which may in turn enhance its affinity to the cognate receptor molecule, as previously reported [42], [43], [44]. Thus, it is not surprising that a prolonged half-life and better antitumoral activity were observed. Future studies will be focused on pharmacokinetics on non-human primates. In addition, 6 histidine residues are included in the carboxyl terminus of rhEndostatin, which are used for affinity purification. At present, we are not sure whether the addition of these residues would affect the activity of rhEndostatin.

Previous studies showed that endostatin has an additive effect with chemotherapy agents towards various types of tumors [45], [46], [47] with little toxicity [5], [7], [12]. Endostar has been approved for clinical trials in China. Preclinical studies indicated that the combination of Endostar with chemotherapy is more effective to inhibit lung tumor growth and metastases [48], [49]. Most recently, clinical data from multiple institutes suggest that the strategy to combine Endostar and chemotherapy agents can dramatically improve outcomes of cancer patients [50], [51], [52]. Similar results have been observed in our preclinical models that the rhEndostatin exhibits a potent antitumor activity against homogeneic and allogeneic tumors in mice alone or in combination with CTX, as well as DDP. The commercially available Endostar is also included in our study. The tumor inhibition rate by Endostar and rhED are about 22% and 28%, respectively. On the other hand, no significant difference has been found between these two factors. Furthermore, our studies reveal that an enhanced tumor inhibitory activity can be achieved when rhEndostatin is administered peritumorally, implying that the route of drug delivery may affect its bioactivity. It has been shown that endostatin binds to the blood vessels when it is administered systemically, resulting in the decreased angiogenic protein concentration around the tumor microenvironment [53]. Angiogenesis inhibitors are most effective when administered in a way which maintains a stable concentration in the circulation [54]. Local administration of rhEndostatin may help to maintain an effective concentration around tumors.

A combination of endostatin with chemotherapy agents is currently employed to improve the therapeutic efficacy. However, its application is often hampered by either non-overlapping or overlapping toxicities [10], [55]. Moreover, due to unstable genome, tumor cells frequently develop multi-drug resistance to various chemotherapies [56]. While Endostatin exerts a cytostatic effect on endothelium, which is less prone to the development of drug resistance [55], [57]. In the present study, we have demonstrated that a combination of conventional chemotherapy agent, CTX, with rhEndostatin exhibits an additive effect on the metastasis of B16 melanoma. Our research data also confirm that a combination of cytotoxic antitumor drug, DDP, with rhEndostatin results in an additive effect on growth inhibition of A549 xenograft tumors. This combination strategy aims at multiple targets of tumor development, thus enhancing the therapeutic efficacy and reducing the toxicity of chemotherapy and occurrence of drug resistance.

Our current study demonstrates that prokaryotically expressed, soluble rhEndostatin exhibits a potent anti-tumor effect. rhEndostatin that is administered peritumorally achieved a better anti-tumor activity than that achieved by intravenous injection. Moreover, rhEndostatin displays increased tumor inhibition in A549 tumor cell xenografts in mice when it is combined with DDP. Furthermore, rhEndostatin exhibits an additive effect with CTX on inhibition of metastasis of B16 melanoma. Our preclinical studies strongly suggest that rhEndostatin can be further explored for clinical trials for eventual applications in the clinic.

## Supporting Information

Figure S1 **Inhibition of human A549 lung carcinoma growth by administrating rhEndostatin or Endostar.** A549 lung carcinomas were implanted to nude mice at the dosage of 6×10^6^ cells each, subcutaneously. Tumor-bearing mice were treated peritumorally with either Endostar (▴, 5 mg/kg/day) or rhEndostatin (▪, 5 mg/kg/day), for successively 15 days. PBS (⧫)was injected at the same volume as the vehicle control. The tumor volume was measured on day 0, 3, 6, 9, 12, 15 post injections. Results were presented as mean±SD (n = 10, each).
(TIF)

Checklist S1 **ARRIVE Guidelines.**
(DOC)
