# Supplementary figures and images for: Anti-Tumor Effect of a Novel Soluble Recombinant Human Endostatin: Administered as a Single Agent or in Combination with Chemotherapy Agents in Mouse Tumor Models
Source: PLoS One. 2014 Sep 17;9(9):e107823. doi: 10.1371/journal.pone.0107823 (PMC4168263; doi:10.1371/journal.pone.0107823)

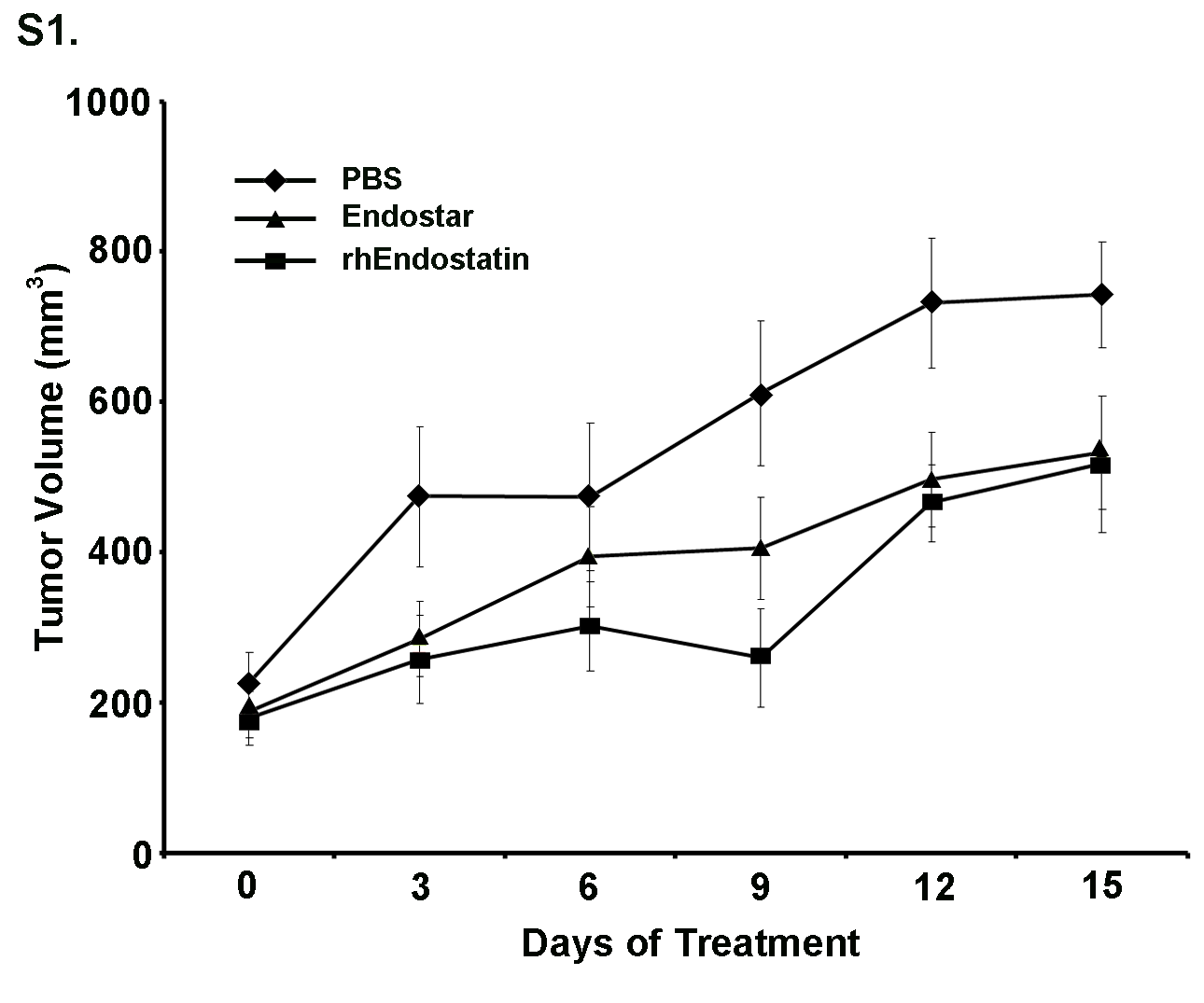

Supplement: Figure S1 — Inhibition of human A549 lung carcinoma growth by administrating rhEndostatin or Endostar. A549 lung carcinomas were implanted to nude mice at the dosage of 6×10^6^ cells each, subcutaneously. Tumor-bearing mice were treated peritumorally with either Endostar (▴, 5 mg/kg/day) or rhEndostatin (▪, 5 mg/kg/day), for successively 15 days. PBS (⧫)was injected at the same volume as the vehicle control. The tumor volume was measured on day 0, 3, 6, 9, 12, 15 post injections. Results were presented as mean±SD (n = 10, each). (TIF) [file pone.0107823.s001.tif]
